# Supplementary material for: RaMBat: Accurate identification of medulloblastoma subtypes from diverse data sources with severe batch effects
Source: Mol Oncol. 2026 Jan 22;20(4):1074–89. doi: 10.1002/1878-0261.70211 (PMC13060657; doi:10.1002/1878-0261.70211)
Supplement: Supplementary file 1 — Table S1. Comparing detailed information for each dataset. Table S2. Computational time comparison of RaMBat and other state‐of‐the‐art methods for MB subtyping. Fig. S1. Comparing RaMBat with state‐of‐the‐art methods for MB subtyping by training on microarray data and testing on an RNA‐seq dataset. Fig. S2. Stability analysis of RaMBat and state‐of‐the‐art methods for MB subtyping across 13 independent test datasets. Fig. S3. Comparing RaMBat with rank‐based ML classifiers across 13 independent test datasets with severe batch effects. Fig. S4. Differential rank gene analysis within RaMBat. [file MOL2-20-1074-s001.zip › Supplementary_material.docx]

**Supplementary Table 1** Summary of MB subgroup distribution in training and independent test datasets. Num, number of samples. MB, medulloblastoma.

| Cohorts | Platform | Num | | | | Total |
| --- | --- | --- | --- | --- | --- | --- |
|  |  | SHH | WNT | Group3 | Group4 |  |
| GSE85217 | Affymetrix Human Gene 1.1 ST Array | 223 | 70 | 144 | 326 | 763 |
| GSE10327 | Affymetrix Human Genome U133 Plus 2.0 Array | 14 | 9 | 10 | 26 | 59 |
| GSE12992 | Affymetrix Human Genome U133 Plus 2.0 Array | 7 | 4 | 8 | 20 | 39 |
| GSE21140 | Affymetrix Human Exon 1.0 ST Array | 29 | 8 | 23 | 35 | 95 |
| GSE30074 | Affymetrix Human Gene 1.0 ST Array | 9 | 2 | 3 | 16 | 30 |
| GSE37382 | Affymetrix Human Gene 1.1 ST Array | 10 |  | 5 | 31 | 46 |
| GSE41842 | Affymetrix Human Gene 1.0 ST Array | 3 | 6 | 2 | 6 | 17 |
| GSE49243 | Affymetrix Human Genome U133 Plus 2.0 Array | 58 | - | - | - | 58 |
| GSE50161 | Affymetrix Human Genome U133 Plus 2.0 Array | 9 | 1 | 2 | 7 | 19 |
| GSE50765 | Affymetrix Human Gene 1.1 ST Array | 10 | - | - | - | 10 |
| GSE62803 | Affymetrix Human Gene 1.1 ST Array | 6 | 5 | 14 | 23 | 48 |
| GSE67850 | Affymetrix Human Genome U133 Plus 2.0 Array | 5 | 1 | 9 | 7 | 22 |
| GSE73038 | Affymetrix Human Genome U133 Plus 2.0 Array | 16 | 10 | 9 | 10 | 45 |
| GSE74195 | Affymetrix Human Genome U133 Plus 2.0 Array | 1 | 1 | 7 | 11 | 20 |

**Supplementary Table 2** Subtype prediction was evaluated using all 13 independent test datasets. The prediction times were as follows: RaMBat, 106 seconds; medulloPackage, 61 seconds; MM2S, 136 seconds; and DCGN, 378 seconds.

|  | RaMBat | medulloPackage | MM2S | DCGN |
| --- | --- | --- | --- | --- |
| Subtype prediction | 106 | 61 | 136 | 378 |

**Supplementary Fig. S1** The RNA-seq dataset was derived from OpenPBTA ^43^. State-of-the-art methods include medulloPackage, MM2S, DCGN, and seven ML classifiers. RaMBat achieved the highest performance with 95% accuracy. MB, medulloblastoma.

**Supplementary Fig. S2** Error bars show the standard error calculated from 10 iterations of random sampling (80% training data). (**A**) Overall accuracy of RaMBat, medulloPackage, MM2S, DCGN and other 7 ML classifiers were compared across all 13 independent datasets. Comparative performance of RaMBat, medulloPackage, MM2S, DCGN and other 7 ML classifiers based on (**B**) GSE73038, (**C**) GSE10327, (**D**) GSE21140, (**E**) GSE67850, (**F**) GSE50765, (**G**) GSE49243, (**H**) GSE30074, (**I**) GSE37382, (**J**) GSE74195, (**K**) GSE62803, (**L**) GSE41842, (**M**) GSE50161, (**N**) GSE12992 in terms of accuracy, respectively. MLP, multilayer perceptron; KNN, k-nearest neighbors; XGBoost, eXtreme gradient boosting; RF, random forest; LR, logistic regression; SVM, support vector machine; MM2S, medullo-model to subtypes. MB, medulloblastoma; ML, machine learning. Accuracy was reported as mean values across n = 10 independent repetitions of random 80% training data subsampling, with error bars representing the standard deviation (SD).

**Supplementary Fig. S3** (**A**) Models trained on the full training set and evaluated on all 13 test datasets. (**B**) Repeated random sampling of 80% of the training samples (10 iterations with different seeds), with error bars estimated across runs to evaluate stability. ML, machine learning.

**Supplementary Fig. S4** (**A-F**) The differentially ranked gene for specific medulloblastoma subtypes. The x-axis represented the gene rank difference where y-axis showed -log10(p-value). Red dots indicated significantly up-ranked genes and blue dots represented significantly down-ranked genes. Triangles represented top 5 up/down-ranked genes. Differentially ranked genes between SHH and WNT (**A**), SHH and Group 3 (**B**), SHH and Group 4 (**C**), WNT and Group 3 (**D**), WNT and Group 4 (**E**), Group 3 and Group 4 (**F**).
