# Supplementary figures and images for: RaMBat: Accurate identification of medulloblastoma subtypes from diverse data sources with severe batch effects
Source: Mol Oncol. 2026 Jan 22;20(4):1074–89. doi: 10.1002/1878-0261.70211 (PMC13060657; doi:10.1002/1878-0261.70211)

**A**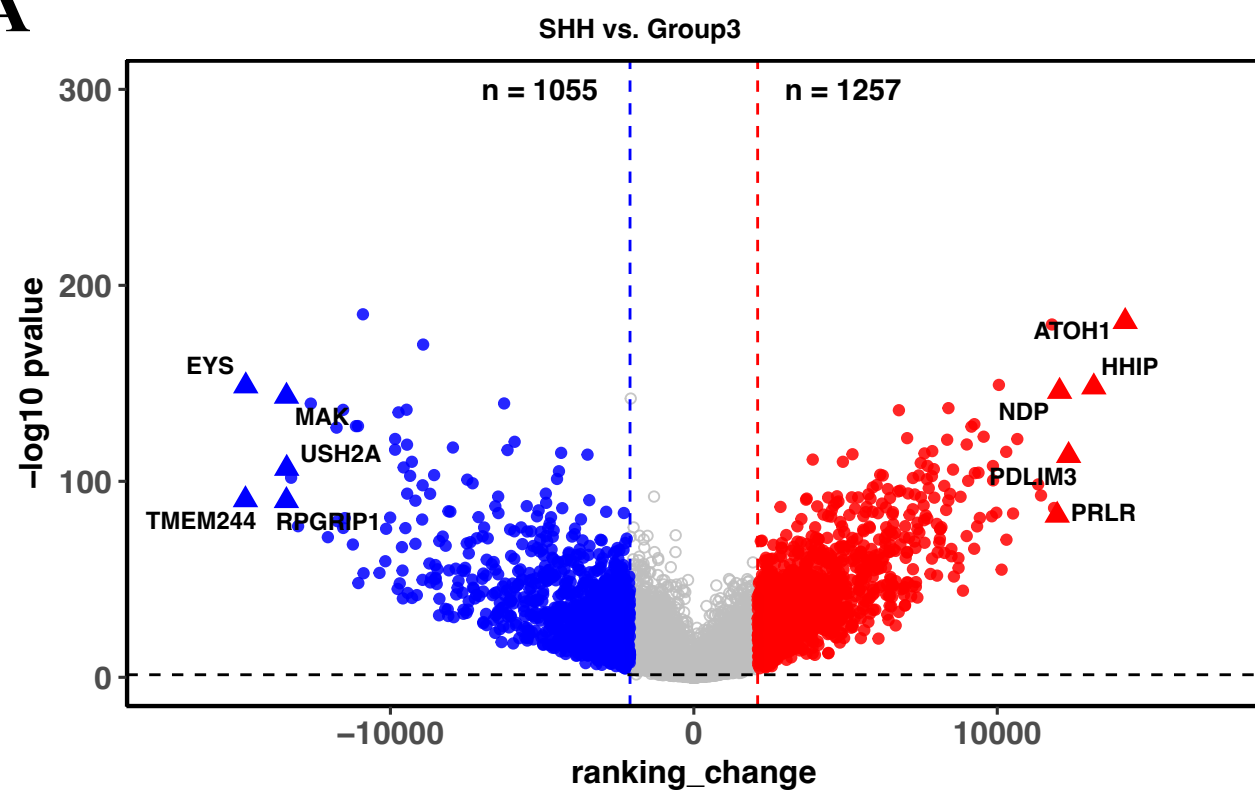**B**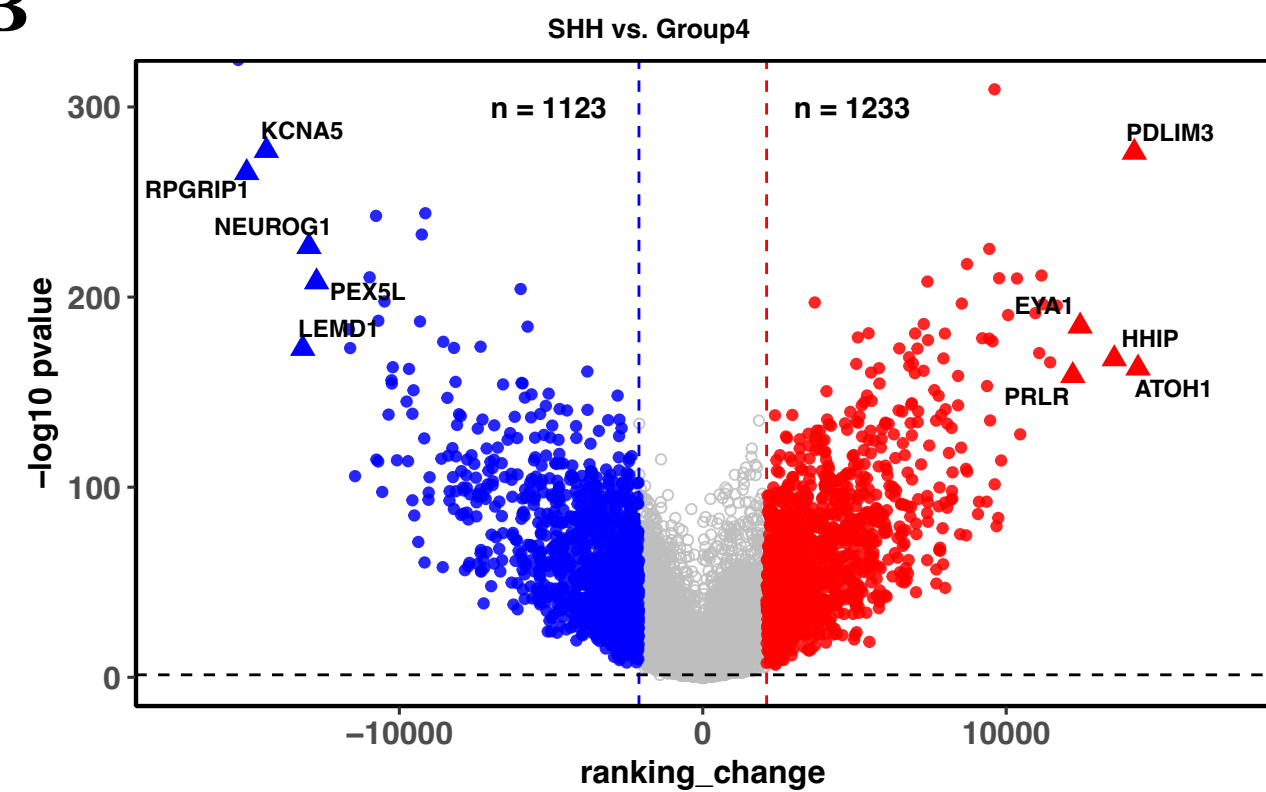**C**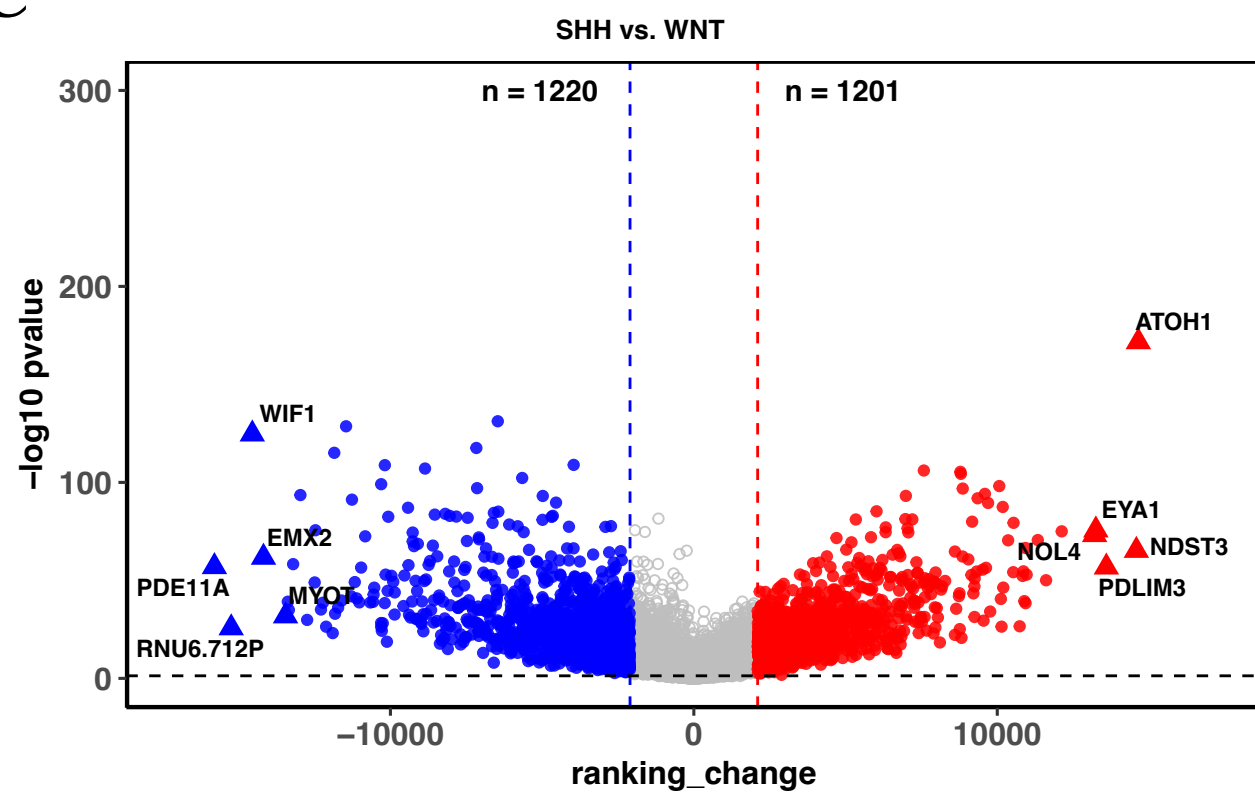**D**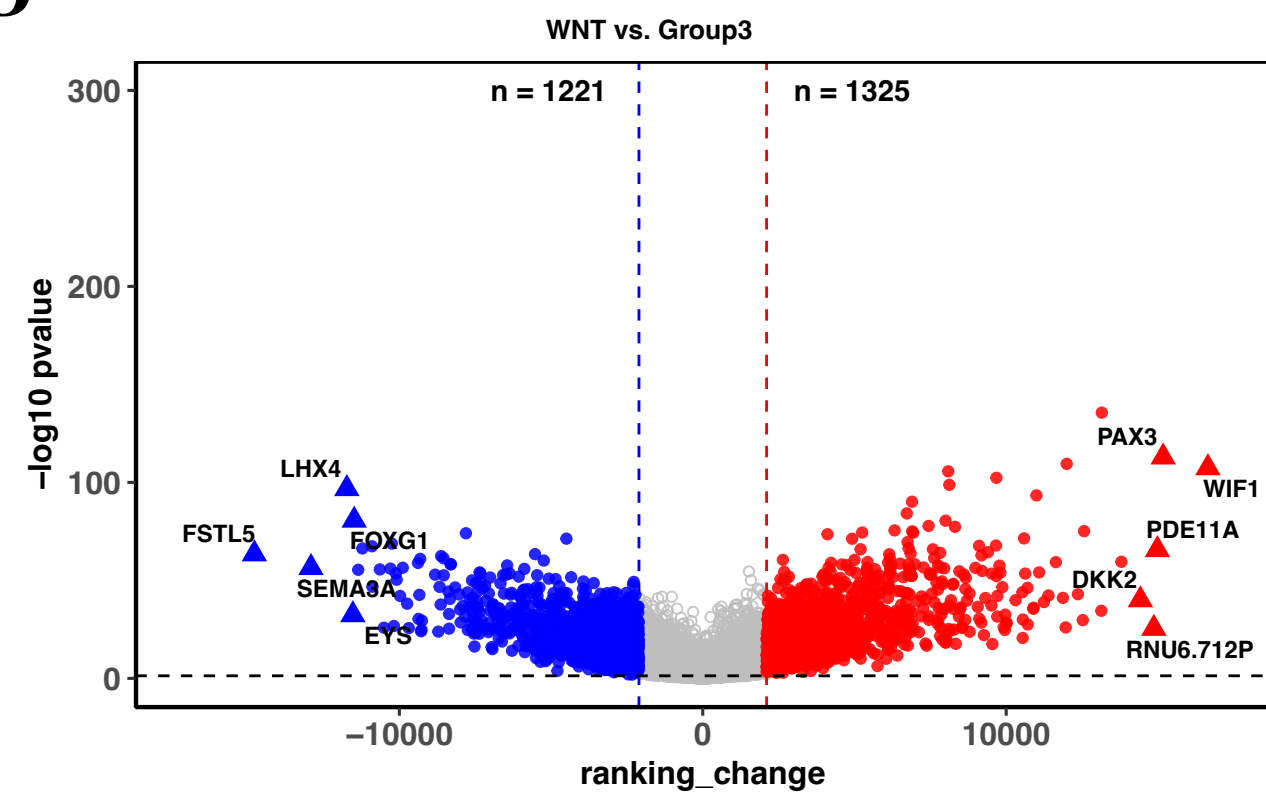**E**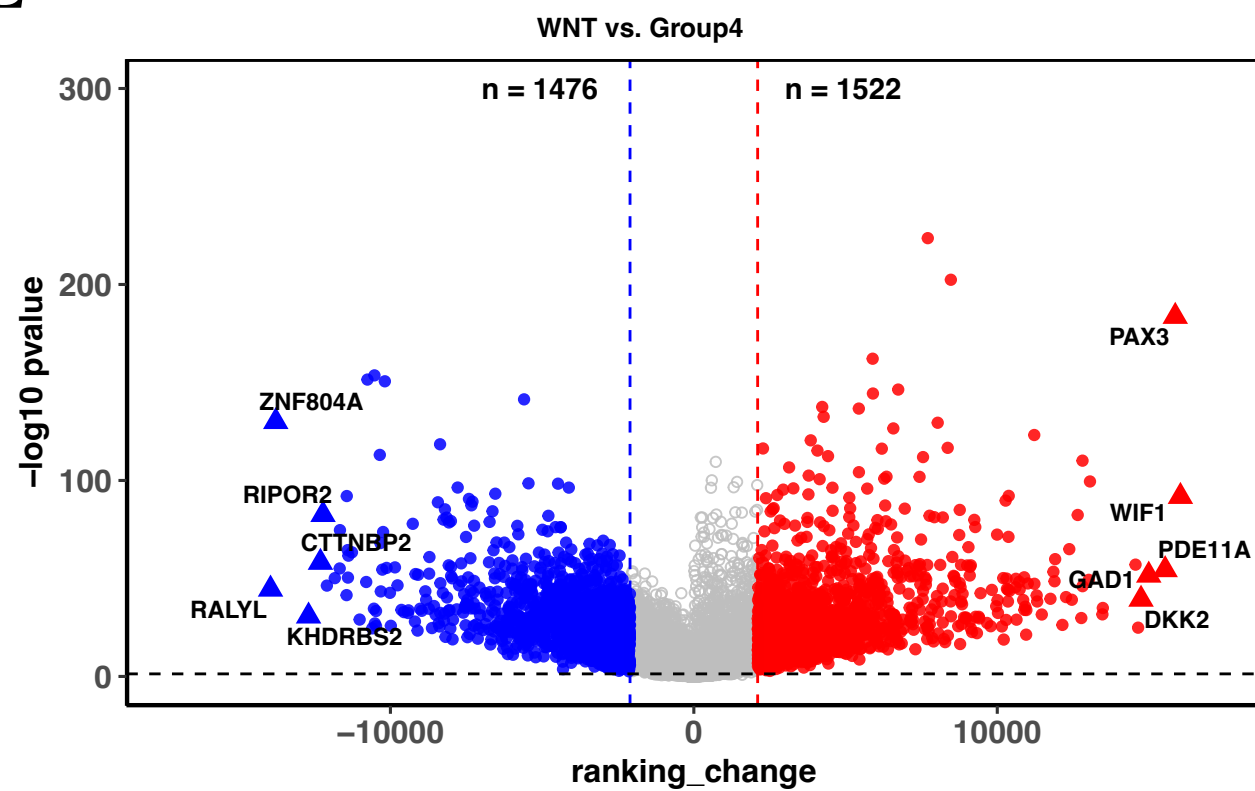**F**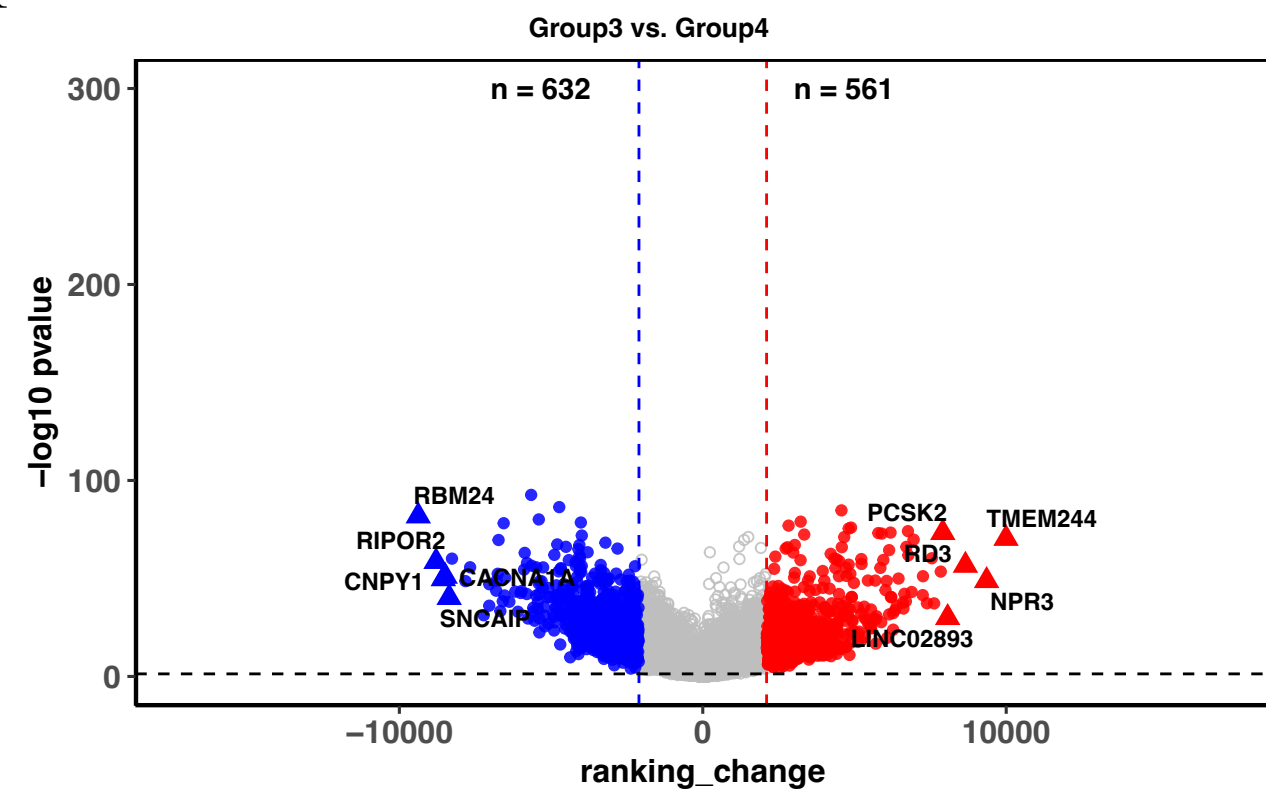

Supplement: Supplementary file 1 — Table S1. Comparing detailed information for each dataset. Table S2. Computational time comparison of RaMBat and other state‐of‐the‐art methods for MB subtyping. Fig. S1. Comparing RaMBat with state‐of‐the‐art methods for MB subtyping by training on microarray data and testing on an RNA‐seq dataset. Fig. S2. Stability analysis of RaMBat and state‐of‐the‐art methods for MB subtyping across 13 independent test datasets. Fig. S3. Comparing RaMBat with rank‐based ML classifiers across 13 independent test datasets with severe batch effects. Fig. S4. Differential rank gene analysis within RaMBat. [file MOL2-20-1074-s001.zip › Supplementary Fig. S4.pdf]

# RNA Dataset

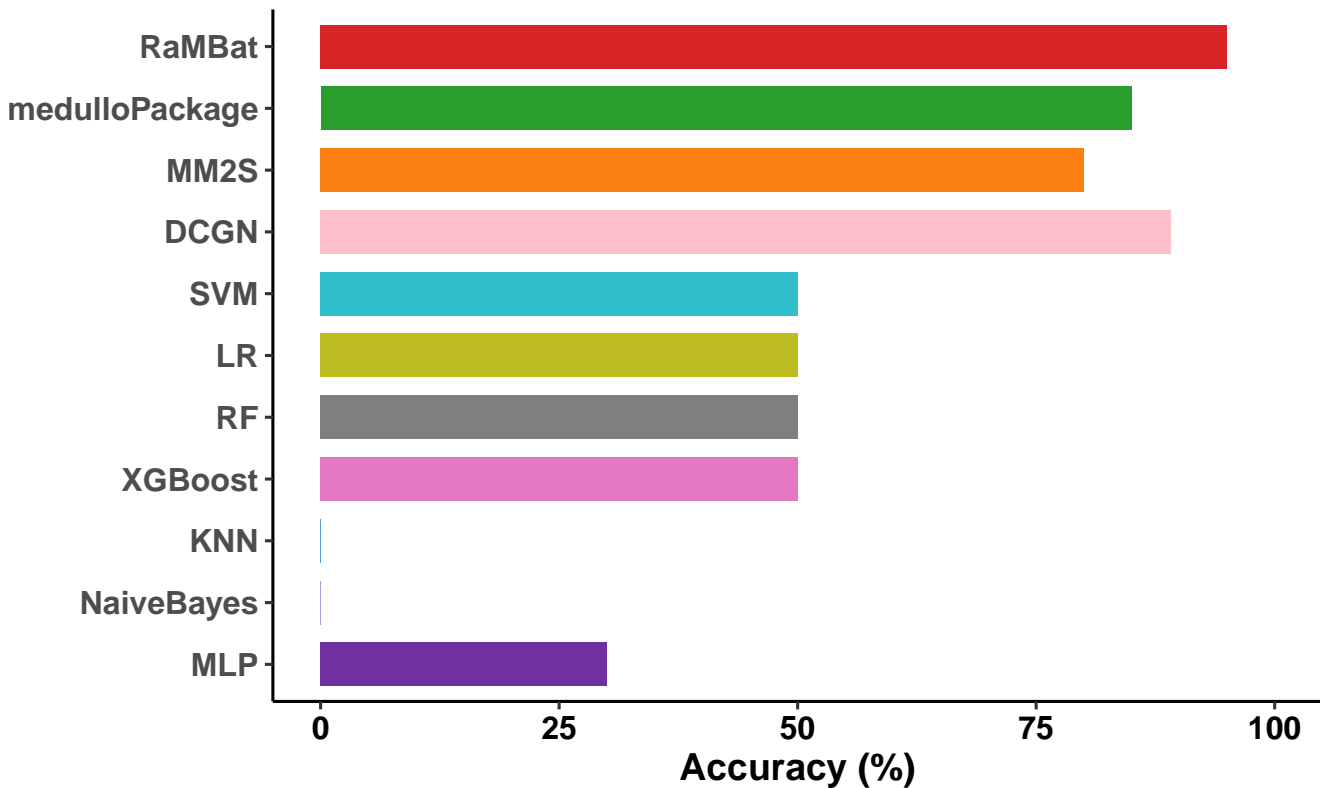

Supplement: Supplementary file 1 — Table S1. Comparing detailed information for each dataset. Table S2. Computational time comparison of RaMBat and other state‐of‐the‐art methods for MB subtyping. Fig. S1. Comparing RaMBat with state‐of‐the‐art methods for MB subtyping by training on microarray data and testing on an RNA‐seq dataset. Fig. S2. Stability analysis of RaMBat and state‐of‐the‐art methods for MB subtyping across 13 independent test datasets. Fig. S3. Comparing RaMBat with rank‐based ML classifiers across 13 independent test datasets with severe batch effects. Fig. S4. Differential rank gene analysis within RaMBat. [file MOL2-20-1074-s001.zip › Supplementary Fig. S1.pdf]

A

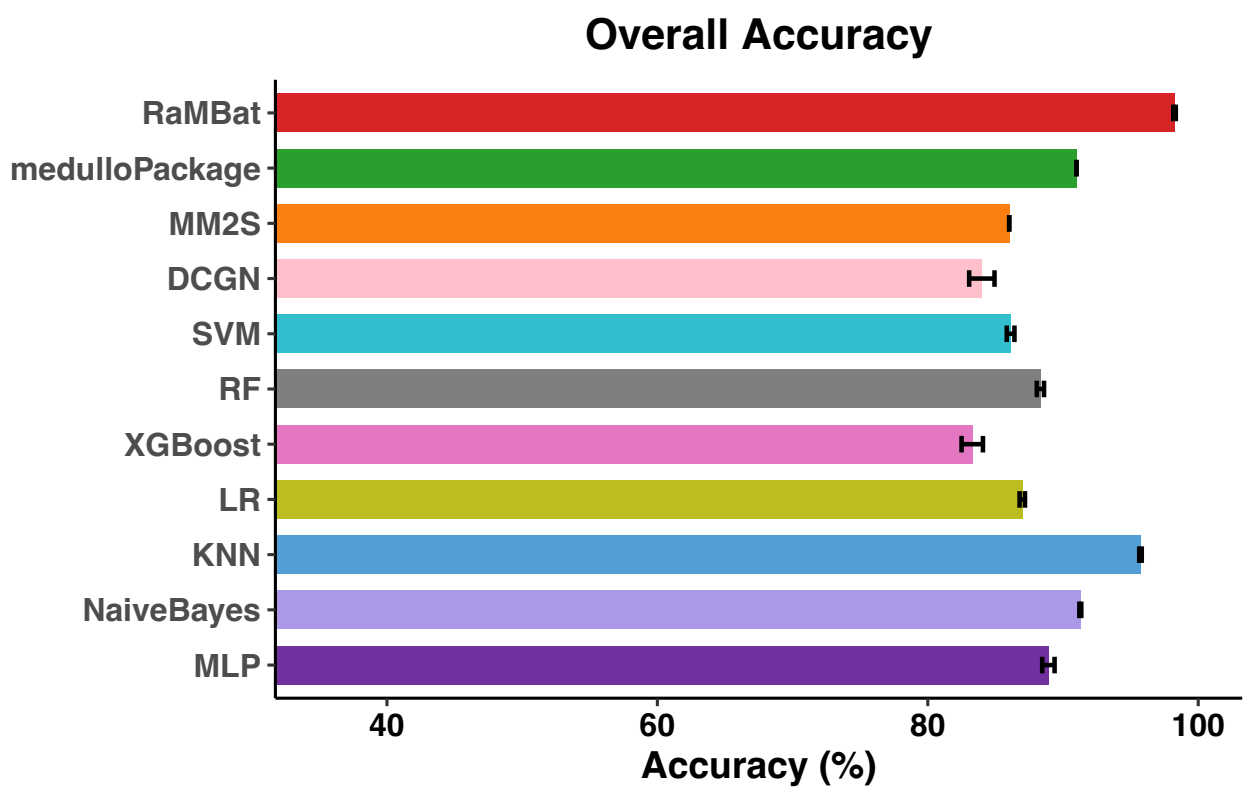

B

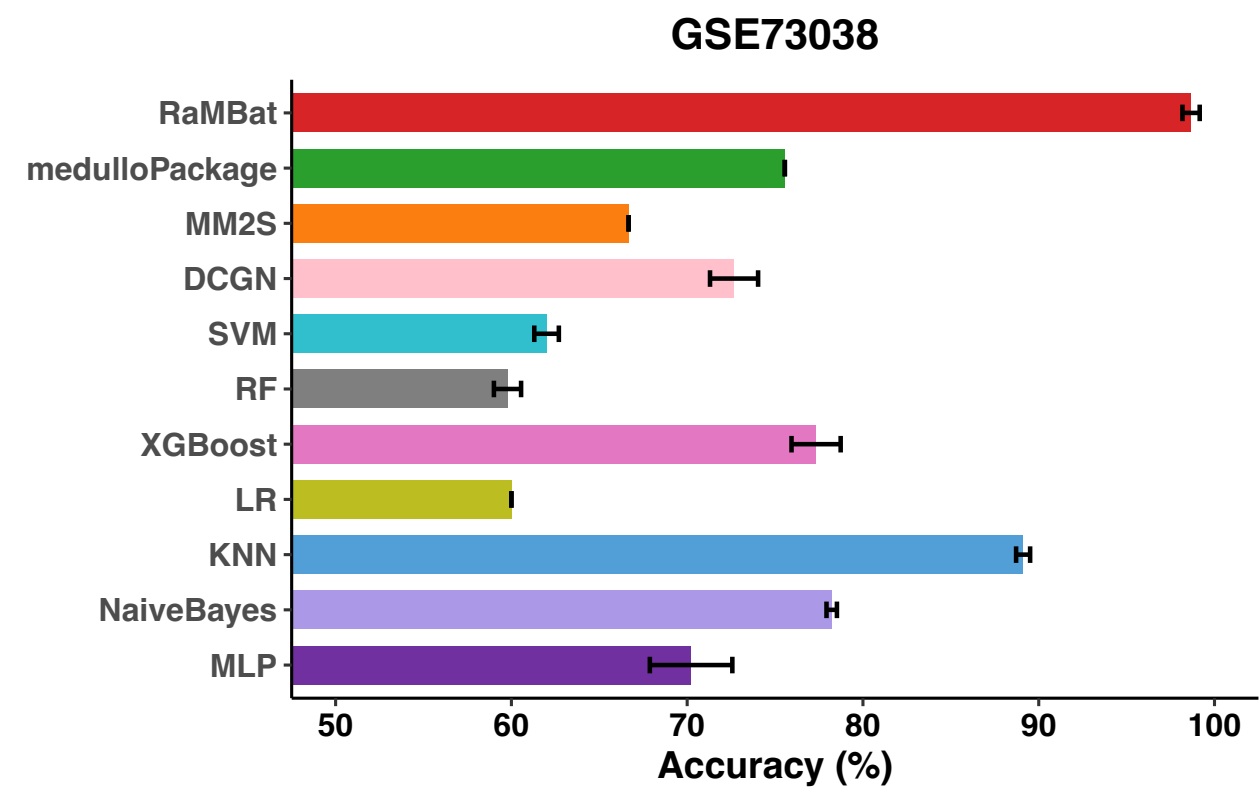

C

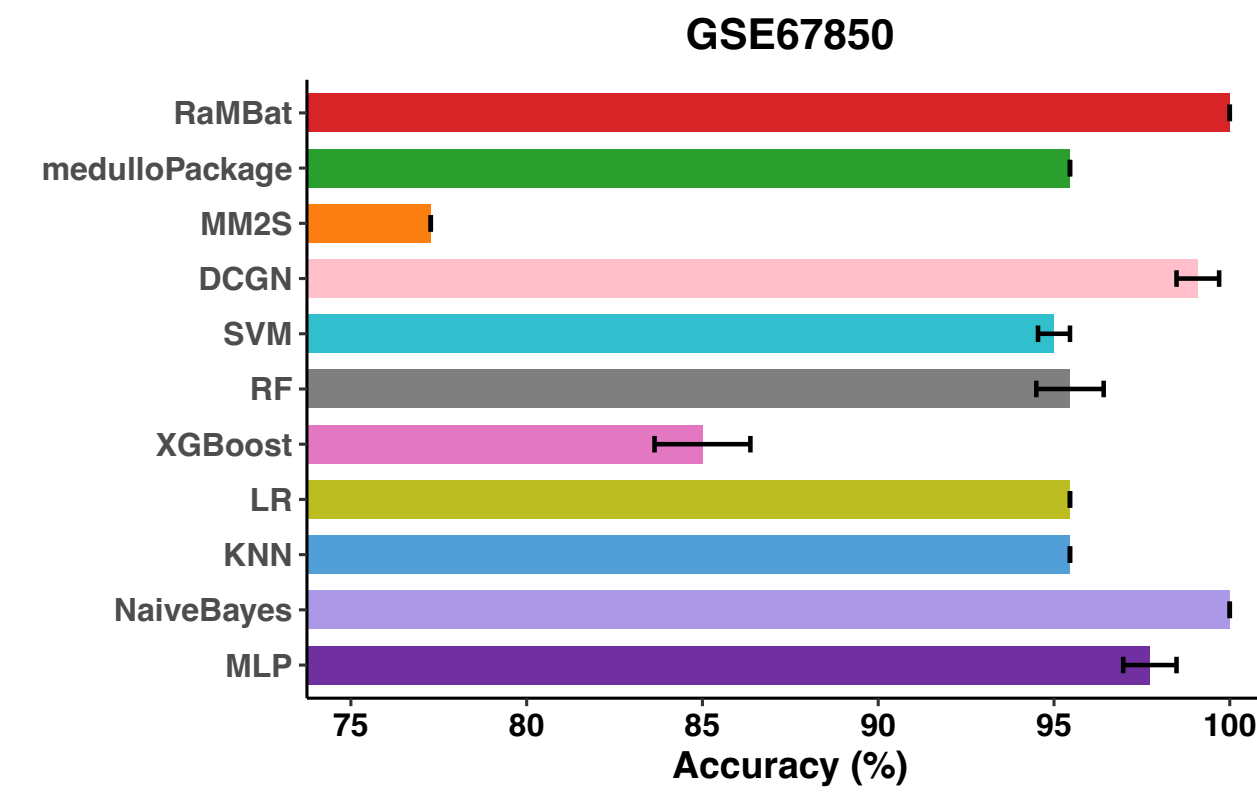

D

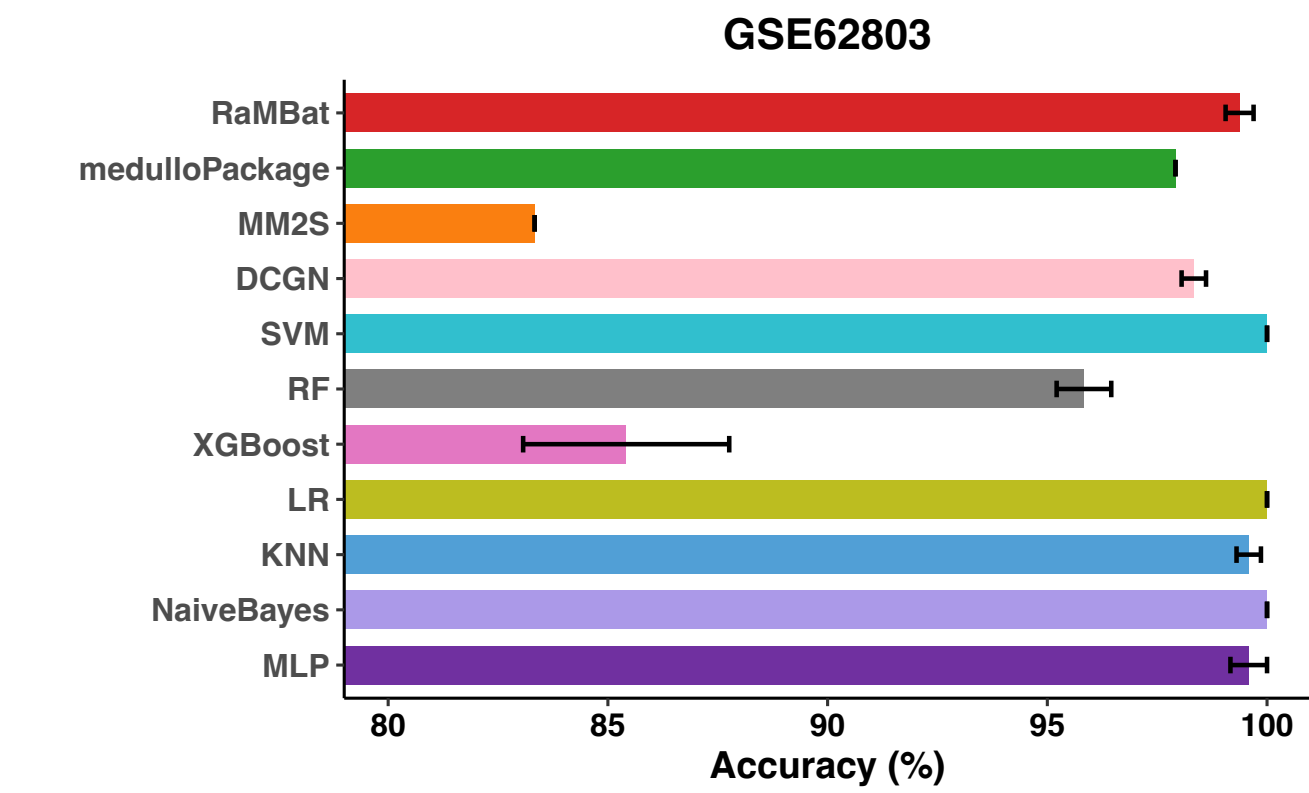

E

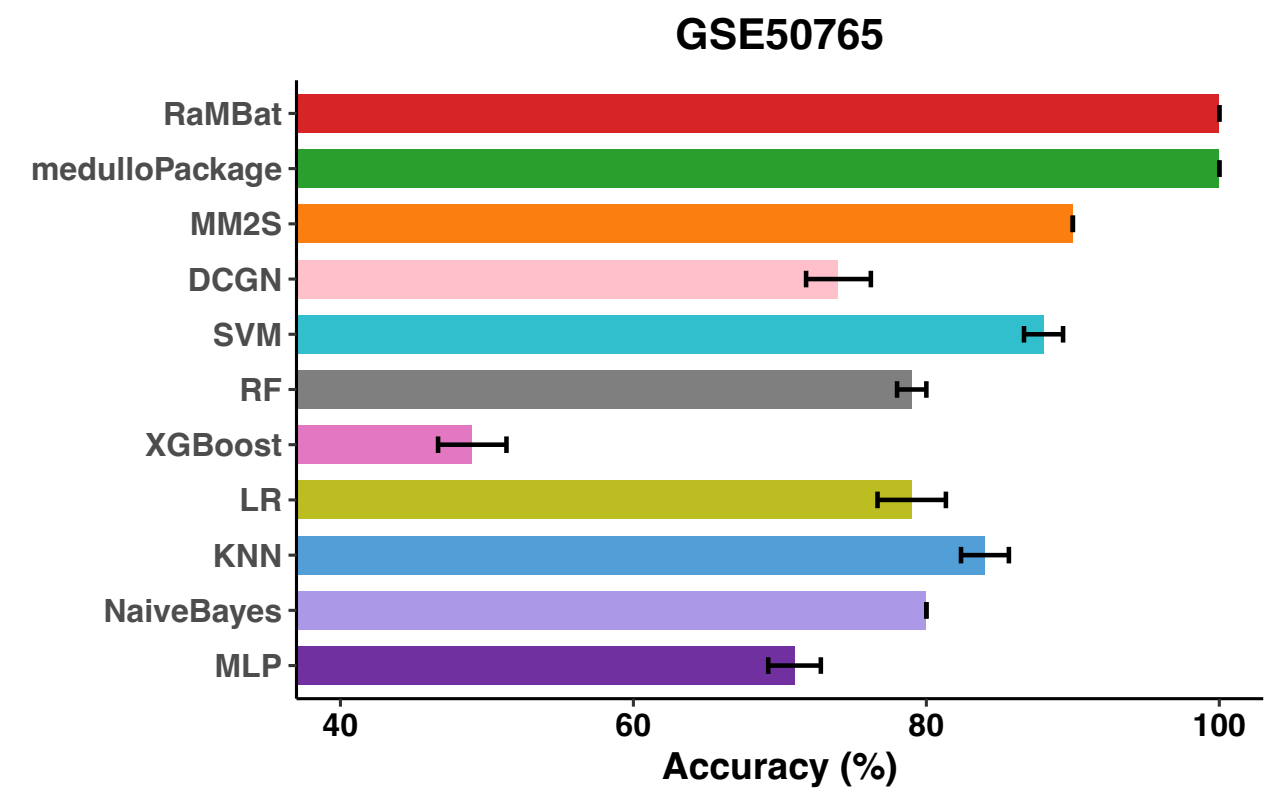

F

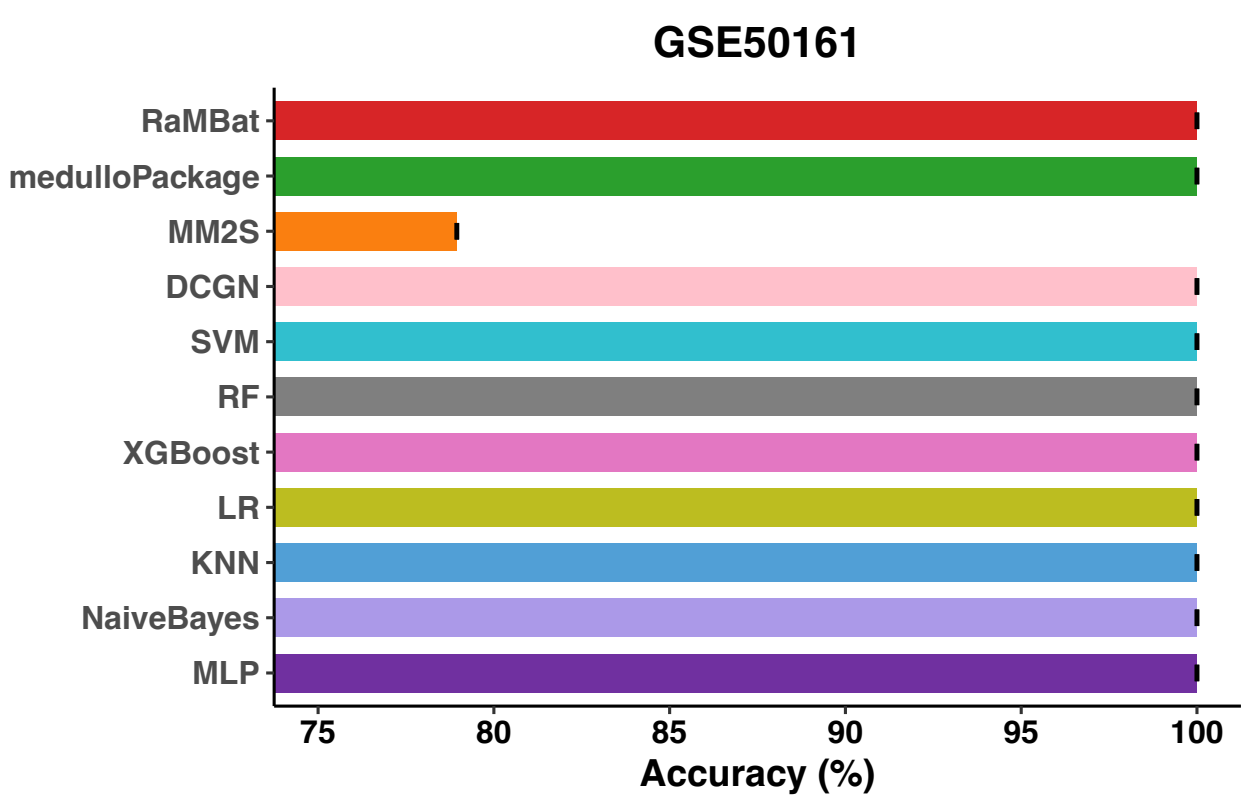

G

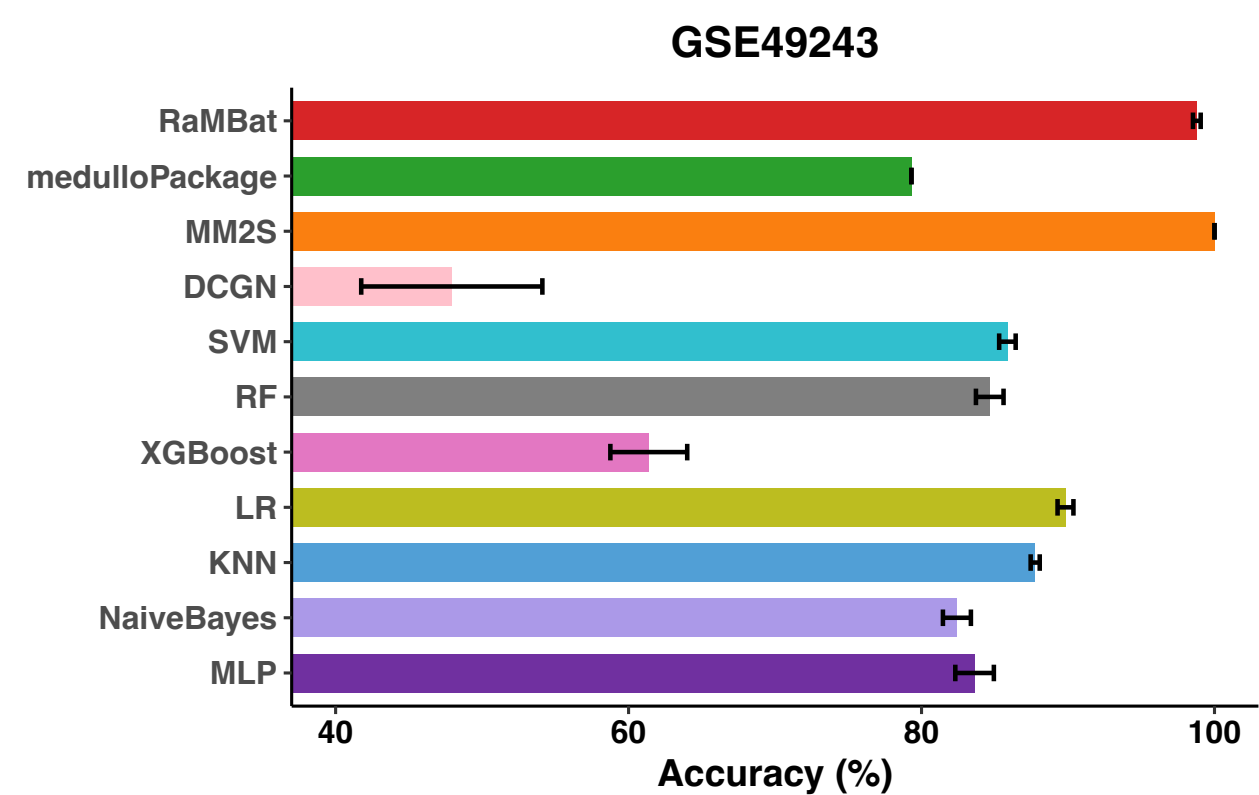

H

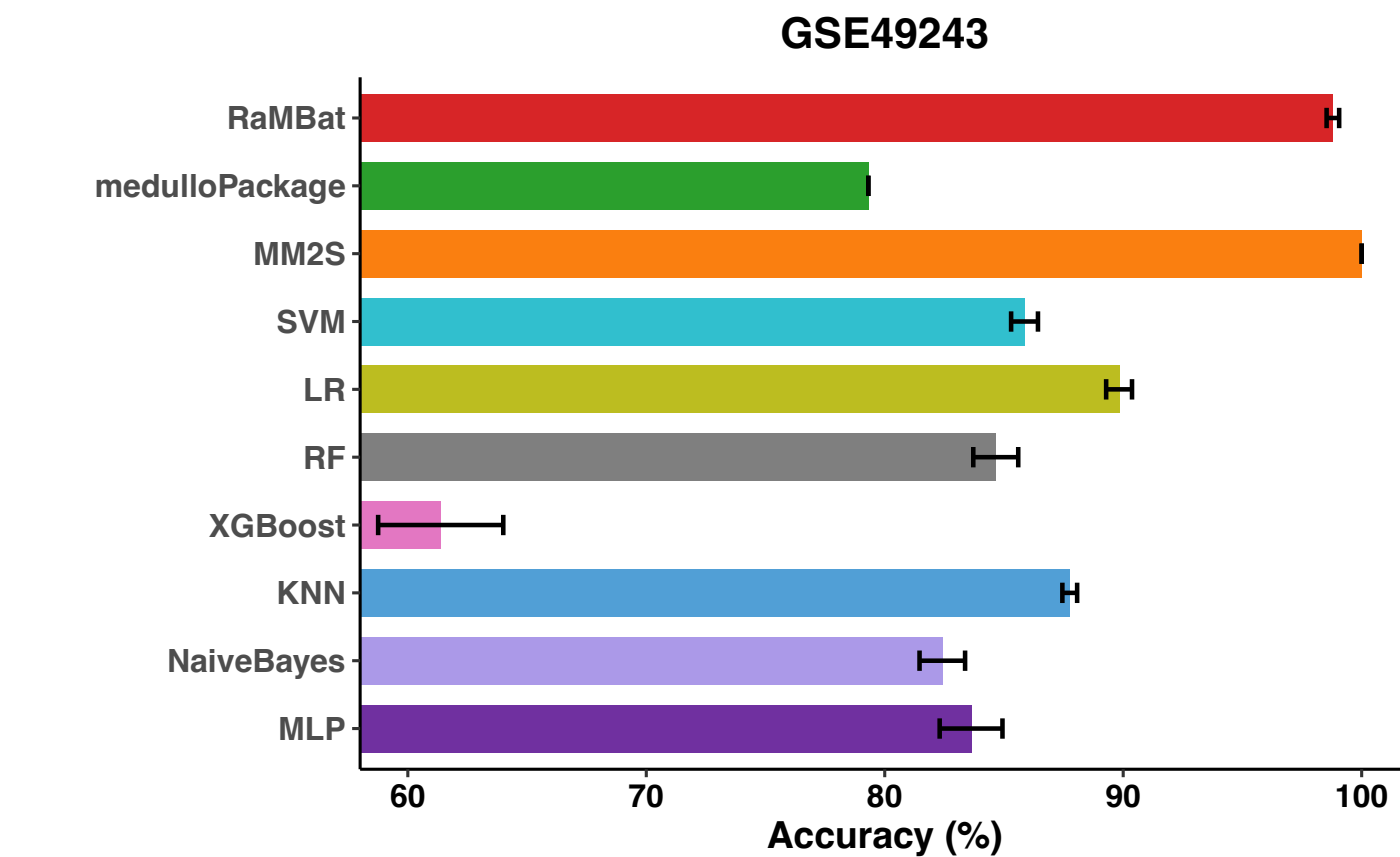

I

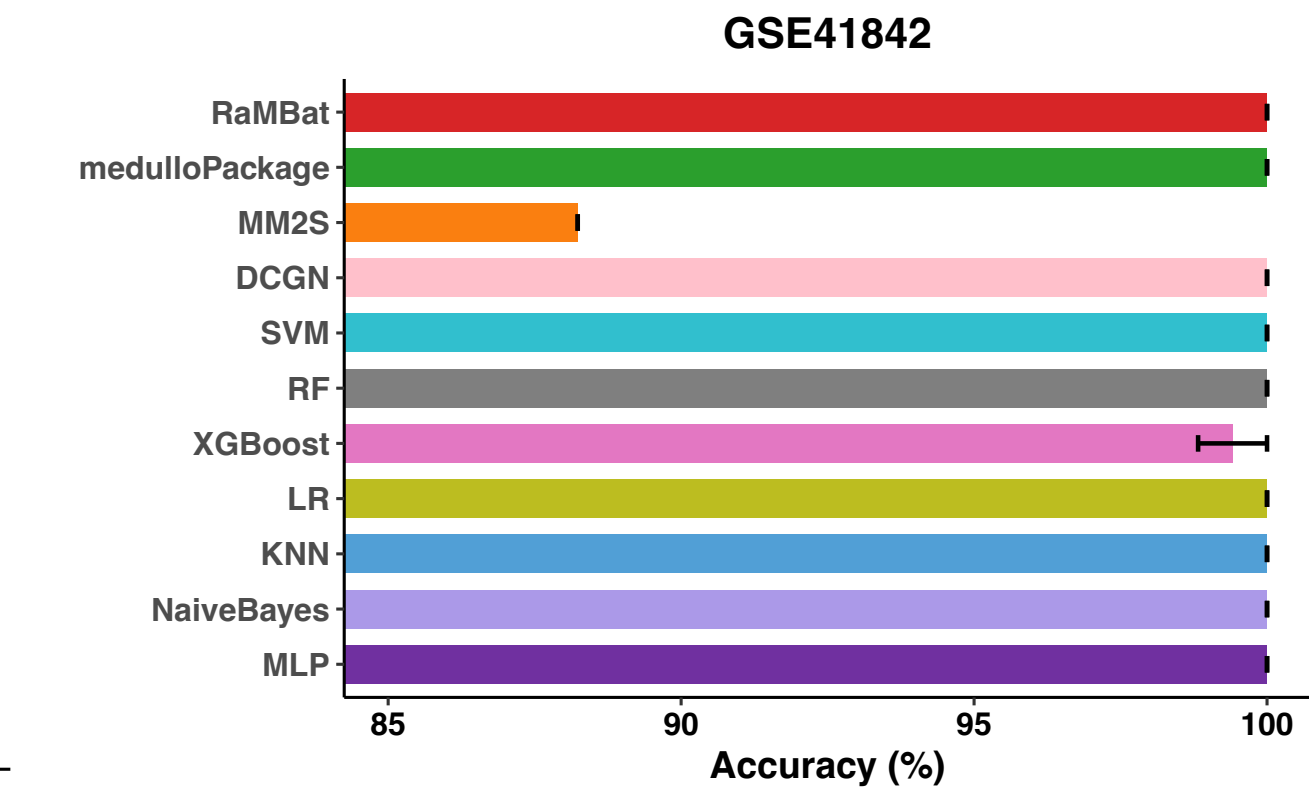

J

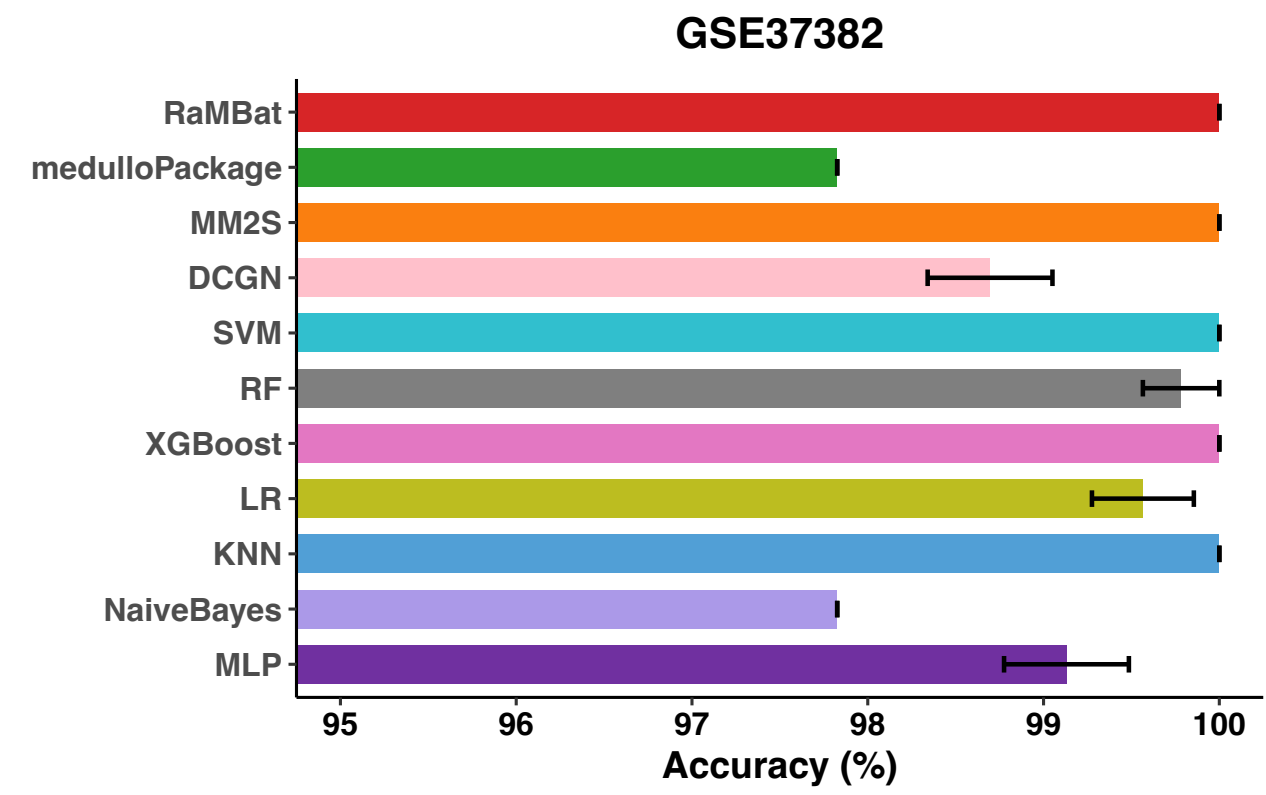

K

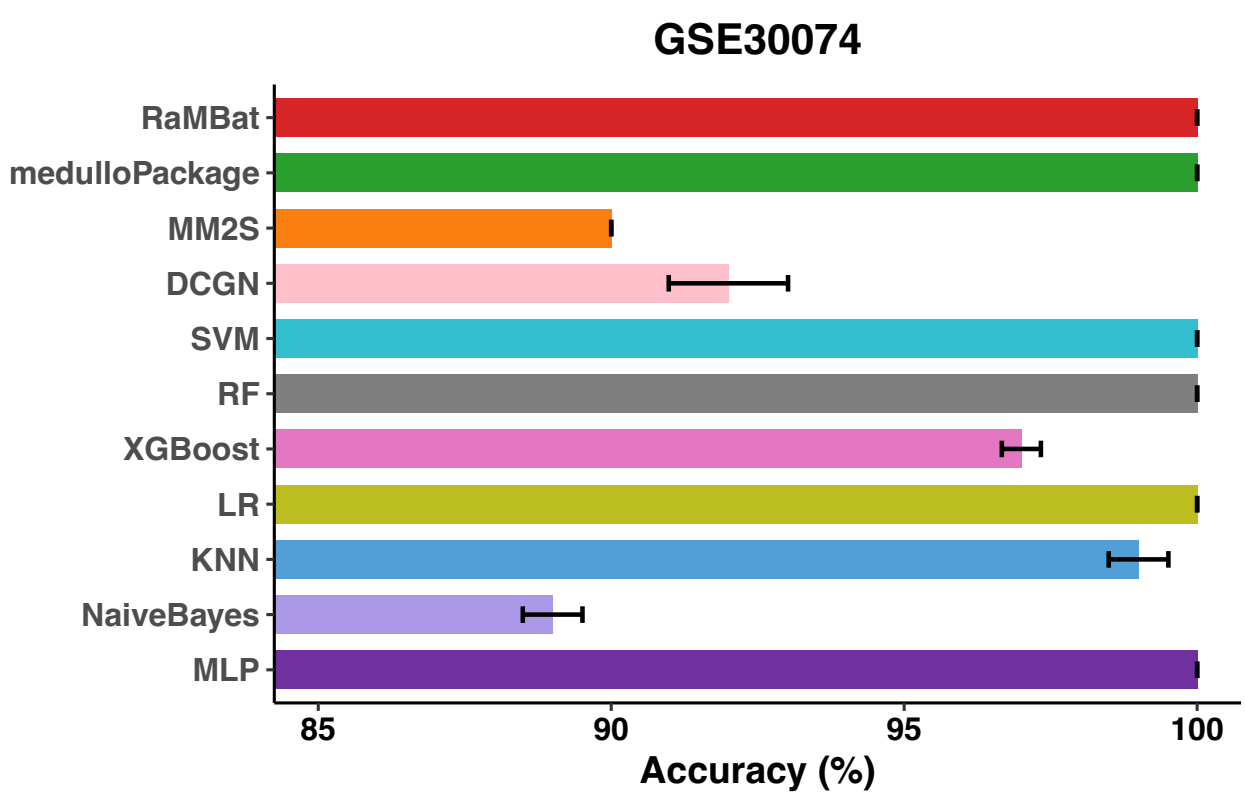

L

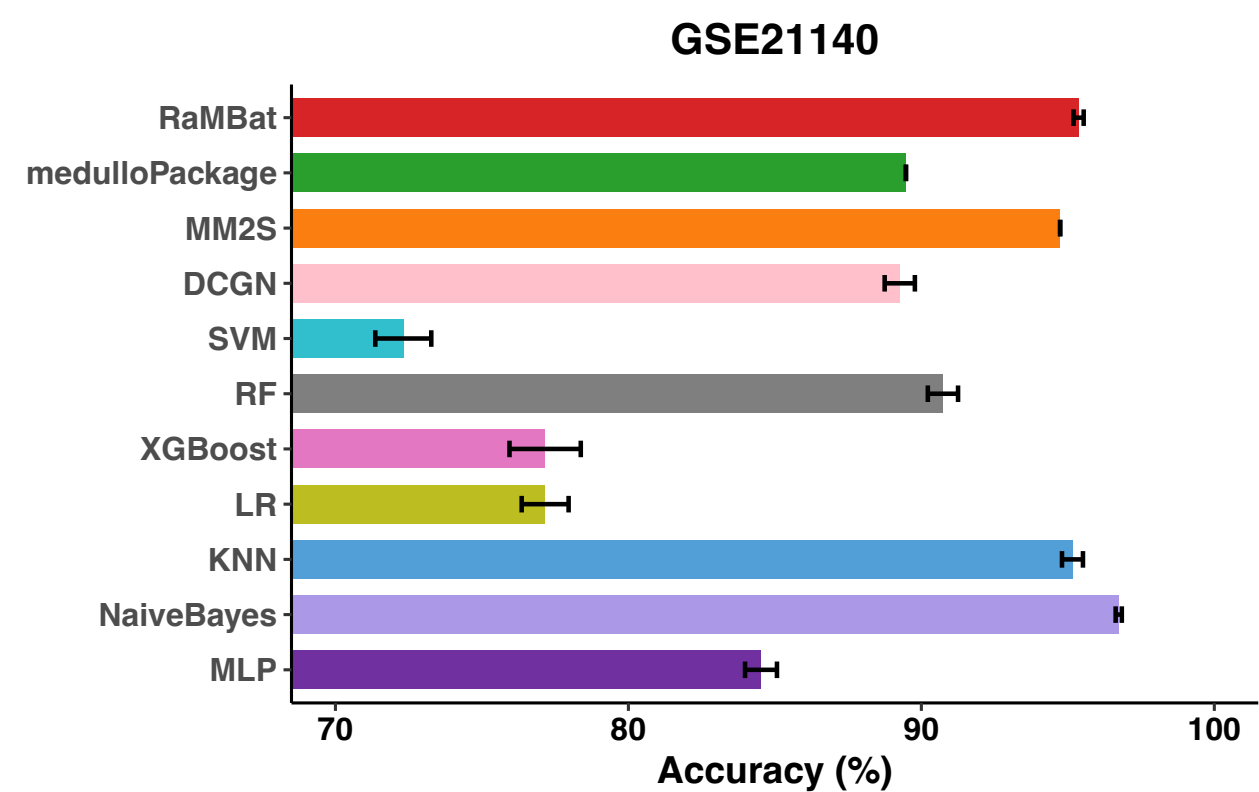

M

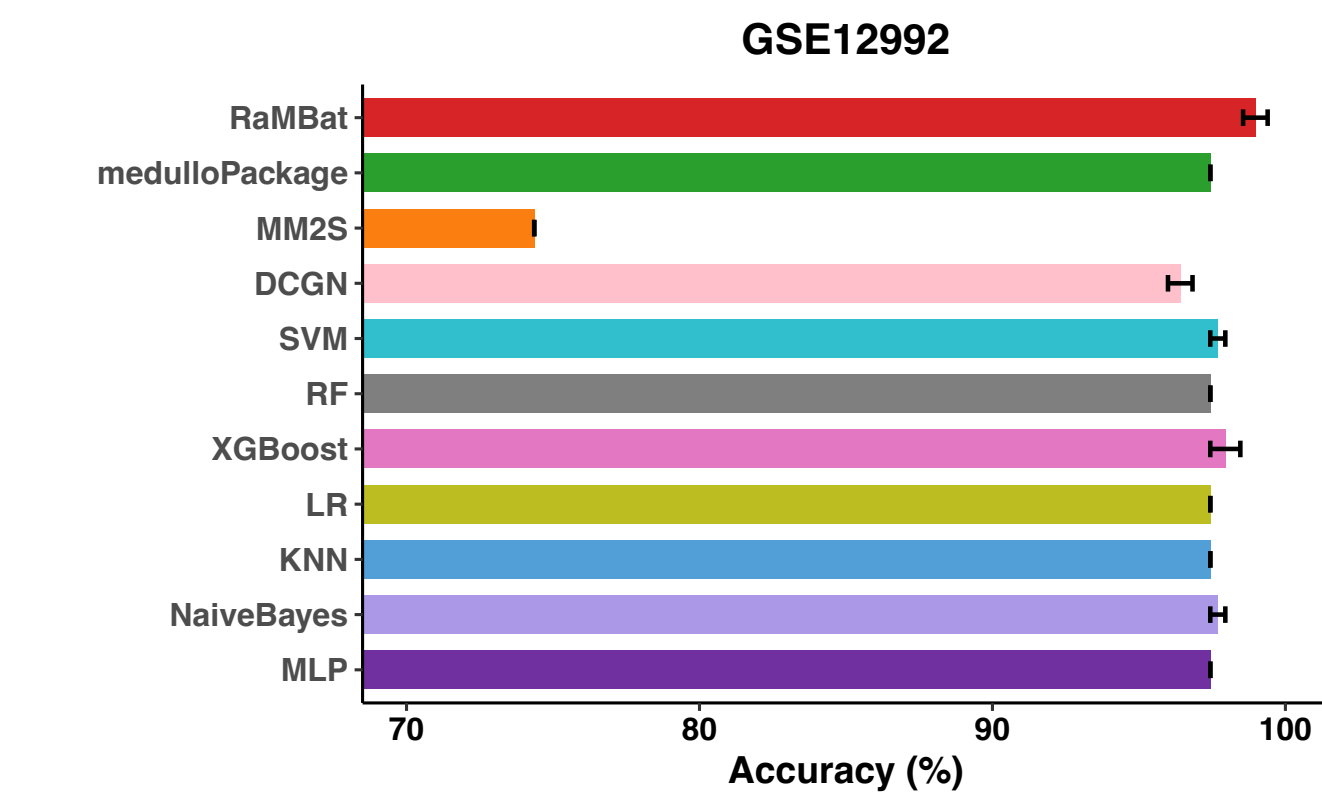

N

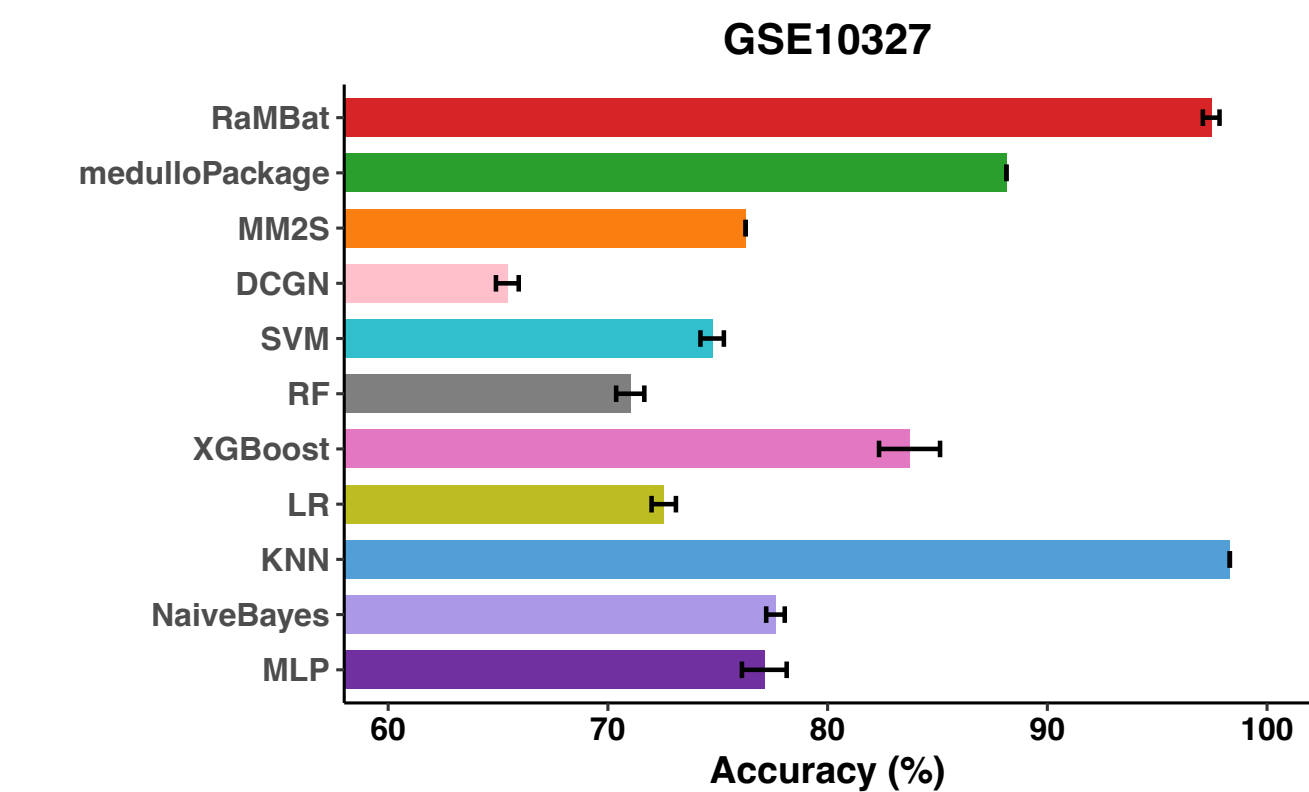

Supplement: Supplementary file 1 — Table S1. Comparing detailed information for each dataset. Table S2. Computational time comparison of RaMBat and other state‐of‐the‐art methods for MB subtyping. Fig. S1. Comparing RaMBat with state‐of‐the‐art methods for MB subtyping by training on microarray data and testing on an RNA‐seq dataset. Fig. S2. Stability analysis of RaMBat and state‐of‐the‐art methods for MB subtyping across 13 independent test datasets. Fig. S3. Comparing RaMBat with rank‐based ML classifiers across 13 independent test datasets with severe batch effects. Fig. S4. Differential rank gene analysis within RaMBat. [file MOL2-20-1074-s001.zip › Supplementary Fig. S2.pdf]

**A****Overall Accuracy**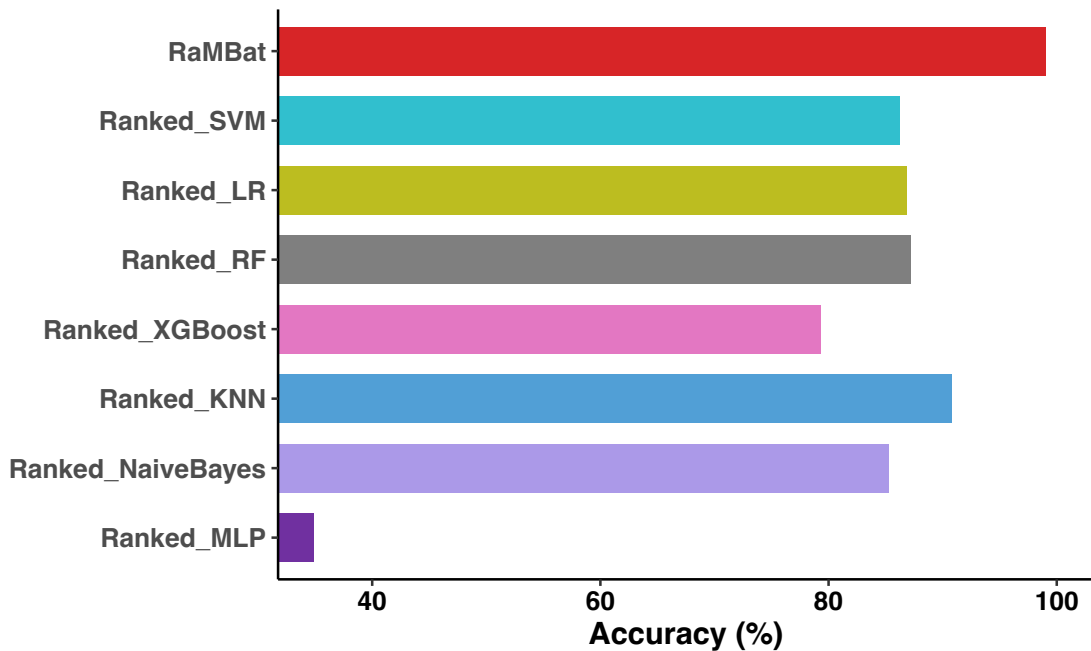**B****Overall Accuracy**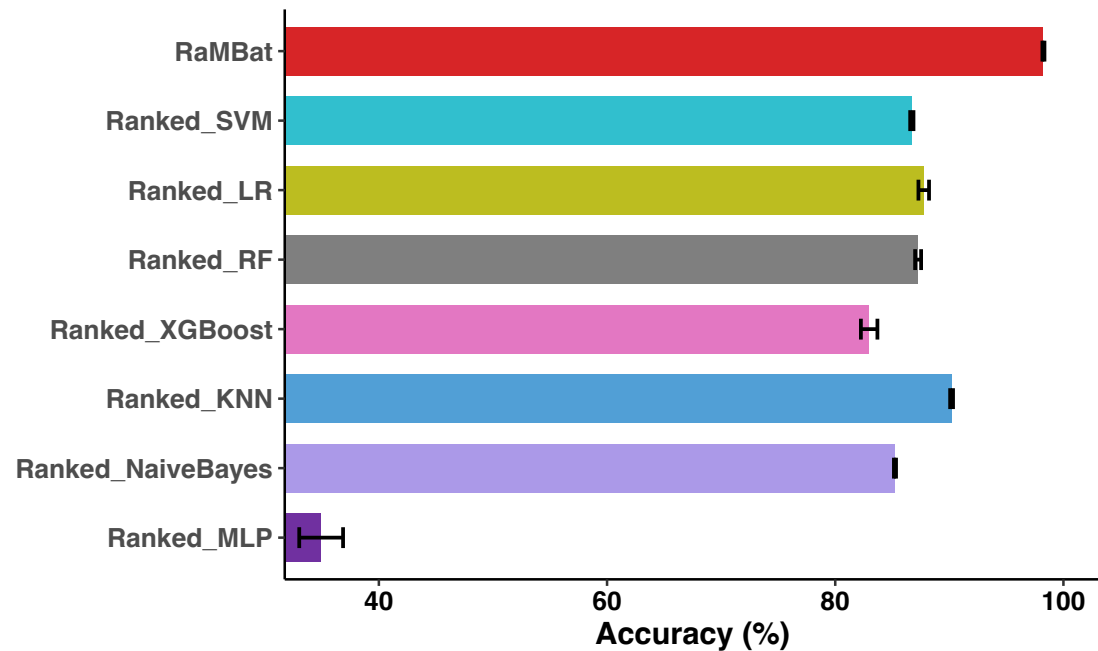

Supplement: Supplementary file 1 — Table S1. Comparing detailed information for each dataset. Table S2. Computational time comparison of RaMBat and other state‐of‐the‐art methods for MB subtyping. Fig. S1. Comparing RaMBat with state‐of‐the‐art methods for MB subtyping by training on microarray data and testing on an RNA‐seq dataset. Fig. S2. Stability analysis of RaMBat and state‐of‐the‐art methods for MB subtyping across 13 independent test datasets. Fig. S3. Comparing RaMBat with rank‐based ML classifiers across 13 independent test datasets with severe batch effects. Fig. S4. Differential rank gene analysis within RaMBat. [file MOL2-20-1074-s001.zip › Supplementary Fig. S3.pdf]
